# Jervis Street Hospital

**Published:** 1840-01-01

**Authors:** 


### JERVIS STREET HOSPITAL.

### DR. CORRIGAN ON THE TREATMENT OF ACUTE RHEUMATISM BY OPIUM.*

Dr. Corrigan has written a very long, and a very interesting series of Clinical Observations on this subject. We shall mark out the main passages, and present them to our readers.

Dr. Corrigan remarks, that it is a desideratum to find a plan of treatment, which will shorten the duration of the disease—prevent the occurrence of the serious complications of pericarditis, endocarditis, &c. and not reduce the strength of the patient. He believes, that the treatment by opium will be found to possess the advantages sought for. It shortens the duration of the disease; it enables the patient to pass through so painful a disease with comparatively little suffering; it husbands his strength, so that when convalescent he rises from bed with comparatively little depression of vascular energy and muscular strength; and lastly, it diminishes very remarkably the tendency to the occurrence of such complications.

After relating eight cases, Dr. Corrigan observes :—

"The most important rule to be remembered in employing opium for the cure of acute rheumatism is, *that full and sufficient doses* shall be exhibited. I have heard of opiate treatment having disappointed some who have tried it. On inquiry, I have learned that in those cases it has been given only to the extent of a grain every fourth or every sixth hour. This is not 'the treatment of rheumatism by opium;' it is making the patient worse than before; it is inflicting on the patient the mischief arising from the stimulant effects of the drug, and withholding from him all the benefits of its sedative influence. The opium should always be increased in dose, both in frequency and quantity, until the patient feels decided relief; and should be then kept up at that dose until the disease is steadily declining. The first indication that tells the practitioner, he has reached the proper dose, is the statement of the patient, who in reply to an inquiry as to how he has passed the night, probably says that he has not slept, but that he is free from pain and feels comfortable. This effect having been attained, the opium may then be continued in repetitions of the same dose as to frequency and quantity. Clarke's Case, No. VIII. shows the rapidly good effects of the large doses. A relapse set in: he got two grains of opium every third hour, uninterruptedly for twenty-four hours. He took sixteen grains of opium within twenty-four hours, and the relapse was suddenly cut short. In Rooney's case, No. III. in the first day eight grains of opium were administered. The dose was then increased to twelve grains within the twenty-four hours for the second, third, and fourth days; and on the fifth day he was taking bark. In Dr. Aldridge's Case, the quantity taken in a fortnight amounted to about 200 grains. I think about ten or twelve grains in every twenty-four hours, will be found the average quantity required. The tolerance of the remedy is a remarkable feature in the treatment, and may, I think, be fairly adduced as an argument in favour of its propriety. The head is not affected by the large quantity

---

* Dublin Journal, Nov. 1839.

of opium administered. This is remarkably shown in Dr. Aldridge's Case, (Case VI.) where the head was not injured by the opium, even though there had been previously a tendency to derangement of cerebral functions in all previous febrile affections. There is another singular circumstance connected with the exhibition of the opium. It is the occurrence of diarrhœa while the patient is using the opium even in full doses; in some instances, the diarrhœa becoming so troublesome as to require starch enemata, or chalk mixture, with kino. It is seldom necessary to purge the patient while administering the opium; indeed the pains are sometimes brought back by the administration of a purgative, either from the patient catching cold in rising from bed, or from the irritability produced by the action of the purgative. The patient's bowels, if they have not been constipated in the commencement of the attack, may be not only safely, but with benefit, not disturbed more than once in two days.

In most cases, Dr. Corrigan applies embrocations to the affected joints—warm spirits of turpentine, or camphorated spirit or simple decoction of poppy-heads, or, when only slight stiffness with little or no swelling remains, a liniment of equal parts of ol. terebinth, and ol. camphorat. with one drachm of sulphur to each ounce of the liniment.

"There arises in the progress of a case of acute rheumatism a stage that is sometimes perplexing. The fever is very much abated, the skin is covered with almost constant perspiration, even sometimes to the degree of producing a miliary eruption, and so profuse in quantity that the patient, when the clothes are raised, steams like one in a vapour bath, and the skin becomes clammy, pale, and soddened; the pains become erratic, and the pulse becomes quicker and smaller: in such a stage, and with these symptoms, the conjunction of sulphate of quinine with opium is the combination that, I think, will be found most beneficial. K.'s Case (No. V.) is an example of such as I have described, and shows the good result of the exhibition of the combination of quinine and opium in that particular stage. Another remedy of considerable efficiency, when the acute stage is passing away, is the mist. guaiaci. The preparations of guaiacum have been an old and favourite popular remedy in rheumatism, and have fallen, perhaps undeservedly, into comparative disrepute. As it is not my wish to travel beyond the acute form of the disease, I shall make no observation on the use of the hydriodate of potass, or of any of the remedies adapted to rheumatism when passing into a chronic state. There is one form of acute rheumatism in which the opiate treatment will cause disappointment, should the practitioner trust to it alone. It is that form which we sometimes meet with, in persons whose habits of living, and perhaps hereditary tendency, give a predisposition to gout, and in whom, when rheumatism does appear, it is not genuine rheumatism, but a combination of gout with rheumatism."

We have long been in the habit of using opium freely in acute rheumatism, but never, we confess, so freely as Dr. Corrigan. His plan is worthy a careful trial, and, if it bears out its author's anticipations, it will be highly valuable.

